# Socio‐Economic Inequalities in Stillbirth and Preterm Birth Rates Across Europe: A Population‐Based Study

**DOI:** 10.1111/1471-0528.18274

**Published:** 2025-07-10

**Authors:** Lucy Smith, Marianne Philibert, Sonya Scott, Maria Jose Vidal Benede, Liili Abuladze, Adela Recio Alcaide, Marina Cuttini, Maria Fernandez Elorriaga, Alex Farr, Alison Macfarlane, Ewa Mierzejewska, Jan Nijhuis, Judith Racape, Jennifer Zeitlin

**Affiliations:** ^1^ Department of Population Health Sciences College of Life Sciences, University of Leicester UK; ^2^ Obstetrical Perinatal and Pediatric Epidemiology Université Paris Cité, Inserm Paris France; ^3^ Public Health Scotland Glasgow Scotland, UK; ^4^ Department of Health Public Health Agency of Catalonia Catalonia Spain; ^5^ Estonian Institute for Population Studies, School of Governance, Law and Society Tallinn University Estonia; ^6^ Instituto de Estudios Fiscales Madrid Spain; ^7^ La Nostra Famiglia Association Eugenio Medea Pediatric Research Hospital Lecco Bosisio Parini Italy; ^8^ Faculty of Nursing, Physiotherapy, and Podiatry Universidad Complutense de Madrid Spain; ^9^ Department of Obstetrics and Gynecology, Division of Obstetrics and Feto‐Maternal Medicine Medical University of Vienna Vienna Austria; ^10^ Centre for Maternal and Child Health Research, School of Medical and Health Sciences, City St George's University of London London UK; ^11^ Institute of Mother and Child Warsaw Poland; ^12^ Department of Obstetrics & Gynaecology Maastricht University Medical Centre, MUMC+ Maastricht the Netherlands; ^13^ School of Public Health Université Libre de Bruxelles Brussels Belgium

**Keywords:** preterm birth, socioeconomic inequalities, stillbirth

## Abstract

**Objective:**

To estimate socio‐economic (SES) inequalities in stillbirth and preterm birth rates across European countries using population‐based routine data.

**Design:**

Cross‐sectional study of national‐level perinatal health and SES indicators (mother's education/occupation or area‐level deprivation).

**Setting:**

Twenty‐four countries in the Euro‐Peristat network.

**Population:**

Seventeen million births in 2015–2019.

**Methods:**

Rates of stillbirth, singleton very preterm birth (VPB) and singleton moderate/late preterm birth (MLPB) were derived from routine national birth data collected with a common protocol.

**Main Outcome Measure:**

Percentage of excess adverse outcomes associated with SES and concentration indices.

**Results:**

Median rates of adverse outcomes were higher in the lowest versus highest SES groups [Stillbirth: 4.9 (interquartile range (IQR):4.30‐5.80)] versus 2.7 (IQR:2.25‐3.14) per 1000 births; VPB: 1.0 (IQR: 0.87‐1.12) versus 0.6 (IQR: 0.59‐0.66) per 100 live births; MLPB: 5.8 (IQR: 5.27‐6.40) versus 4.4 (IQR:4.13‐4.65) per 100 live births. Excess adverse outcomes associated with lower SES varied greatly, particularly for stillbirth (range−3%, 51%) versus VPB (7%, 27%) and MLPB (5%, 20%). Concentration indices further highlighted varying socio‐economic inequalities across countries. Median concentration indices were similar for countries with both lower and higher levels of adverse events, with median CIs of −0.12 for countries with both high and low levels of stillbirth.

**Conclusion:**

We identified widespread but varying inequalities between countries. These seemed to be unrelated to the rate of adverse outcomes. This suggests the need for policy strategies directly targeted to the prevention of stillbirth and preterm birth in low SES populations. Our findings demonstrate the feasibility of monitoring inequalities internationally using routine data to identify effective action.

## 1 Introduction

Perinatal health outcomes such as preterm birth and stillbirth are significant global public health concerns. They have a wide range of psychosocial and health impacts on babies, families and society [1]. Monitoring these health metrics internationally allows understanding of the disparities in healthcare access, quality and outcomes shown to exist between countries [2, 3]. This is vital for benchmarking [4] and the identification and evaluation of effective prevention strategies for improving maternity and neonatal care [5].

Wide inequalities also exist within countries. Socio‐economically disadvantaged groups experience higher rates of stillbirth and preterm birth [6, 7, 8, 9, 10]. These exist irrespective of universal access to health care and social security provision [11] suggesting access to health care alone does not eliminate the impact of socio‐economic inequalities. As adverse perinatal outcomes affect babies and young families and have long‐term consequences on health and life chances, they contribute to the intergenerational transmission of socio‐economic inequalities of health. Therefore, tackling socio‐economic inequalities in perinatal health outcomes has the potential to improve societal health across generations [11, 12].

International comparative studies of socio‐economic inequalities in adverse perinatal outcomes could further aid identification of avoidable causes of mortality. This in turn can inform both policy responses to reduce inequalities within and between countries. Such research is vital to inform policy decisions that can mitigate the long‐standing and pervasive health inequities faced by disadvantaged populations. Consequently, this would ensure that all families, regardless of their socio‐economic status, have equal opportunities for healthy pregnancies and births. The Euro‐Peristat collaboration highlighted widespread and consistent socio‐economic inequalities in stillbirth rates across Europe linked to mothers' educational levels and mothers' and fathers' occupational groups [13]. However, further research has been impeded due to the lack of consistent measures of SES and the complexity of combining the differing measures of SES available internationally.

Here, our overall aim is to investigate socio‐economic inequalities in perinatal health across countries using routine population data and interpretable summary measures which can inform policy initiatives and be used to evaluate them. We firstly explore the availability of measures of socio‐economic position available in routine statistical systems across 30 countries (educational attainment, occupational status or area‐based socio‐economic deprivation). We then assess cross‐country socio‐economic inequalities for stillbirth, very preterm birth and moderate and late preterm birth. Finally, we assess whether inequalities are wider in countries where rates of adverse events are higher.

## 2 Methods

### 2.1 Data Sources

Data were obtained from the Euro‐Peristat project, a collaboration with participation from 30 countries to assess perinatal health in Europe [3, 14]. A comprehensive set of robust comparable indicators of perinatal health outcomes which can be monitored using routine data (10 core and 20 recommended) were defined through Delphi consensus processes [15]. The data collection process was originally through aggregate tables provided by each country and later used a federated protocol for sharing aggregate tables based on an open‐source common data model and R scripts [16]. Data come from national population birth sources, including birth registers, vital statistics and hospital data [17]. As the study is based on routinely collected data aggregated at a national level, ethics approval was not required.

### 2.2 Adverse Perinatal Outcomes

We report on stillbirth (at or after 24^+0^ weeks of gestation, the recommended lower limit for comparable reporting of stillbirth rates in Euro‐Peristat [18, 19]); singleton very preterm birth (≤ 32^+0^ weeks gestation) and singleton moderate and late preterm birth (32^+0^ – 36^+6^ weeks' gestation). Multiple preterm births were excluded due to international differences in rates of multiple birth and their very high rates of preterm birth. Terminations of pregnancy were excluded from stillbirth rates (except in Belgium, Cyprus and the Netherlands but were very rare events ≥ 24 weeks except in Belgium) because of their different aetiology. Poland was only able to provide stillbirth data from 2018 – 2019.

Calculation of rates was based on the following denominators‐stillbirth: total births ≥ 24 weeks; very preterm singleton birth: live singleton births ≥ 22 weeks; moderate and late preterm singleton birth: live singleton births ≥ 32 weeks.

### 2.3 SES Measurement

Mothers' level of education (defined as the highest level of education achieved) was selected as the primary marker of SES, with occupation included where not available [20], as used previously [13]. Data on mother's education were based on the International Standard Classification of Education (ISCED—UNESCO, 1997): 1: none, primary or lower secondary (levels 0–2), 2: higher secondary (level 3) and 3: post‐secondary (levels 4–6). Occupational class was provided for one country (Ireland), coded as 1: no occupation or student, 2: skilled/unskilled workers/technicians/clerical/service occupations and 3: managers/professionals. Where education and occupation were unavailable, births were requested by quintile of nationally defined scores of area‐level deprivation. We aggregated quintiles into three groups of roughly comparable proportions to the average educational group distribution across countries: most deprived 20%, medium deprivation 40% and least deprived 40%.

### 2.4 Statistical Analysis

We described and compared the availability of socio‐economic status (SES) data for all births, stillbirths and preterm births by calculating the proportion of births with missing SES data. We also examined the distribution of births across SES groups in participating countries by calculating the percentage of births within each group. To address missing SES data in our primary analyses, we used a deterministic proportional allocation imputation method. Assuming data were missing at random, we redistributed cases with missing SES information across the three SES categories based on observed distribution patterns within each country‐specific outcome group. This approach maintained relative SES frequencies and preserved national adverse outcome rates.

We applied three approaches to examine the extent of inequalities within and between countries. First, we compared the rates of stillbirth, very preterm singleton birth and moderate and late singleton preterm birth by SES group across countries. Heat maps were used to simultaneously display outcome rates and SES group distributions. We conducted sensitivity analyses by excluding countries with over 20% missing data for a specific outcome and by comparing results from imputed and non‐imputed data sets to assess robustness.

Second, we calculated, for each country, the percentage of excess adverse outcomes attributable to socio‐economic inequality. Using the adverse outcome rate in the highest SES group in each country as a reference, we estimated how many outcomes would occur if this rate applied across all groups. The difference between observed and expected outcomes was then expressed as a percentage of total observed events. To calculate 95% confidence intervals for these socio‐economic gap estimates, we employed a non‐parametric bootstrapping method. We generated 1000 bootstrap samples per country by resampling from binomial distributions based on observed rates, calculating the gap measure in each, and using the 2.5th and 97.5th percentiles to derive confidence intervals. This method captures the uncertainty around both the reference and overall rates without assuming specific data distributions.

Third, we used concentration indices (CIs), derived from the Gini coefficient, to quantify the degree of SES‐related inequality in each adverse outcome [21, 22]. CIs [23] allow comparison across countries with differing SES measures [24, 25, 26] and distributions [27]. We calculated CIs and 95% confidence intervals to represent, in relative terms, the concentration of stillbirth, very preterm birth and late and moderate preterm birth by SES. As shown by the Scottish Public Health Observatory [28] and outlined in Box S1, CIs are visualised as the area between two lines plotting the cumulative population and cumulative adverse outcomes ordered by deprivation. A CI of 0 suggests no inequality, while values range from −1 (higher burden in lower SES groups) to +1 (higher burden in higher SES groups).

For all these statistics, adverse outcome rate, percentage excess outcomes and concentration indices, we reported the median across all countries along with the interquartile range (IQR) and range as robust measures of dispersion.

Finally, we explored whether countries with higher overall adverse outcome rates also exhibited greater inequality. We compared the median, IQR and range of CIs for countries above and below the median adverse outcome rate. Additionally, we used graphical analyses and Pearson correlation coefficients to assess the strength of association between outcome rates and inequality levels.

## 3 Results

### 3.1 SES Data Availability

The number of births, stillbirths and preterm births were available by SES group for 24 out of the 30 European countries participating in the Euro‐Peristat project, representing 17 million total births between 2015 and 2019 (Table S1). Seven countries were unable to provide these data due to lack of available perinatal data (Greece), lack of timely access to data (Austria), lack of SES data in routine birth data (Germany, Iceland) or need for specific linkage for SES data that are not undertaken for the routine data provided to Euro‐Peristat (Sweden and Norway). Seventeen countries provided education data, one provided occupational data (Ireland) and six provided area‐level deprivation data (France, Netherlands, UK and the UK's three smaller devolved nations Scotland, Wales and Northern Ireland). Latvia could provide only two levels of SES due to a lack of differentiation between lower‐ and upper secondary‐level education.

There were low levels of missing SES data for live birth (Table S1) (3.6%; range: 0%–28%) and moderate and late preterm birth (3.6%; range: 0%–30%) but proportions missing were slightly higher for stillbirth (8.4%; range: 0%–63%) and very preterm birth (4.4%; range: 0%–41%). Belgium, Croatia, Czechia and Portugal had over 20% of data missing for one or more outcomes (Table S1).

**FIGURE 1:**
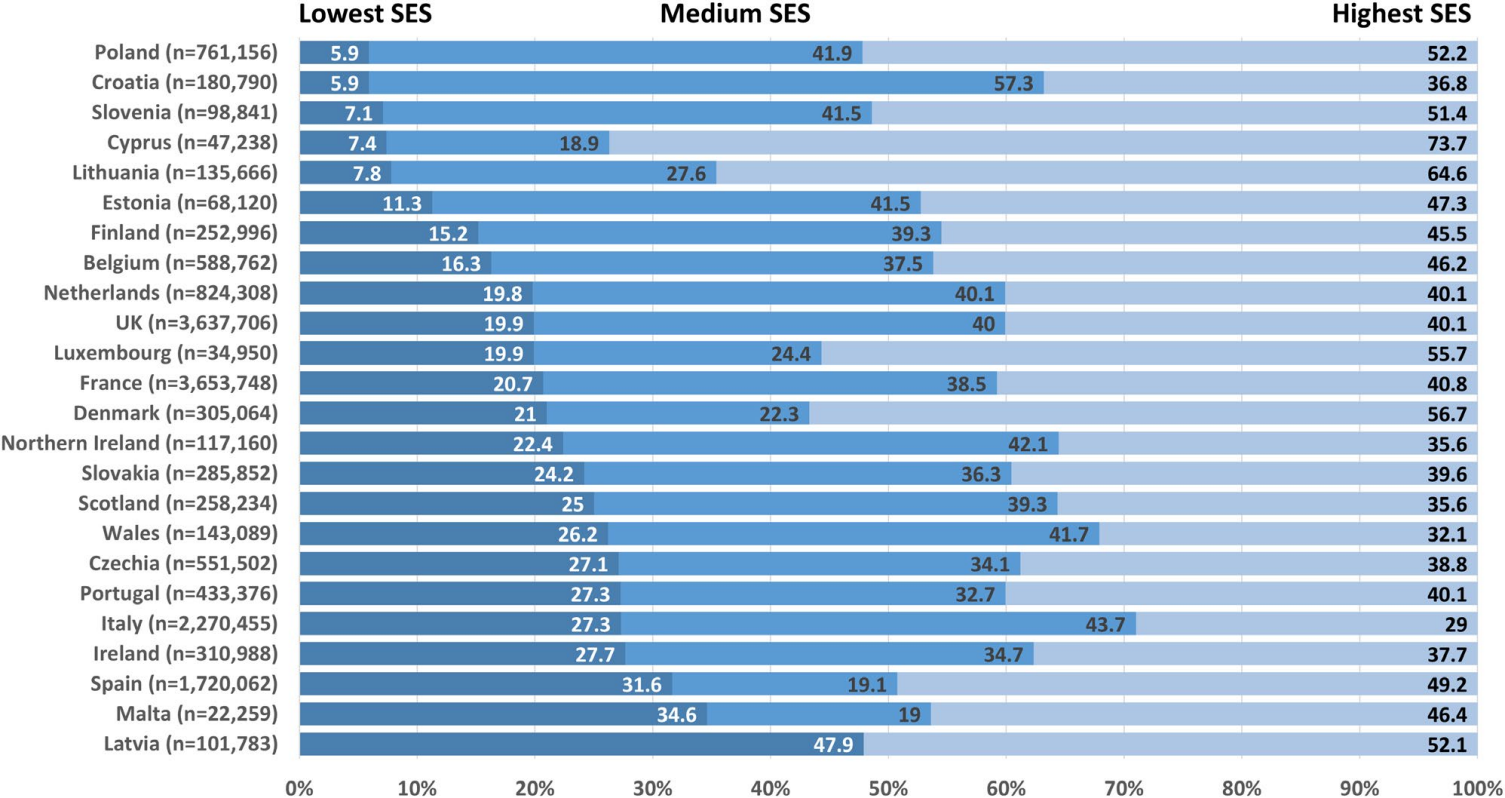
Total number (n) and percentage distribution of livebirths from 24^+0^ weeks gestation by socio‐economic status (SES) level (low, medium and high) for each country.

For countries providing individual SES level based on education or occupation the proportion in the lowest SES group (primary and lower secondary education) ranged between 6% and 48%, the medium SES group (higher secondary) ranged from 19% – 57% and the highest SES (post‐secondary) ranged from 29% – 74% (Figure 1). For countries providing area‐level deprivation scores, these were around 20%, 40% and 40% in the three SES groups, respectively, due to being based on quintiles.

### 3.2 Rates of Perinatal Outcomes by Socio‐Economic Classification

**FIGURE 2:**
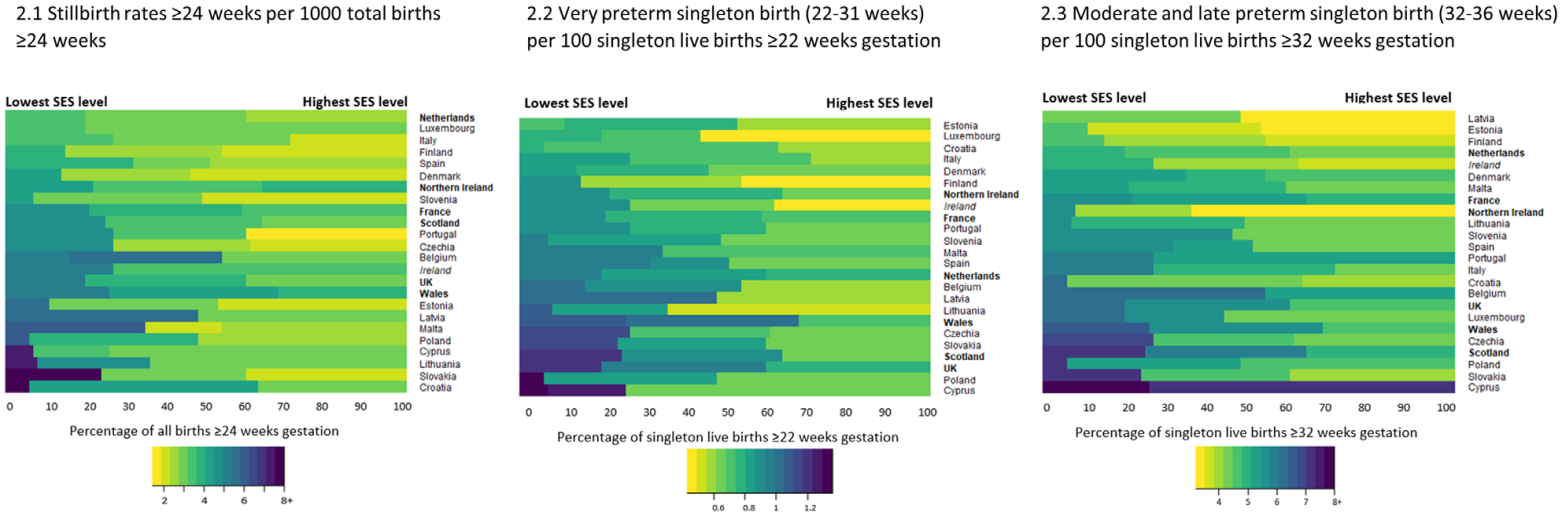
Heatmap of rates of perinatal outcomes by socio‐economic status level and country. Colour key denotes rate of perinatal outcome and the width of the bar represents the proportion of births in that socio‐economic status (SES) level and country names in bold where the measure of SES is based on area‐level deprivation.

Rates of all three adverse outcomes were inversely associated with SES for all countries (Graphically represented in a heatmap in Figure 2 and rates presented in Table S2). Table 1 shows the median rate, IQR and range by SES group for the three adverse outcomes. Across all countries, the median stillbirth rate in the lowest SES group was 4.9 per 1000 total births (IQR: 4.30–5.80), compared to a median of 2.7 per 1000 total births in the highest SES group (IQR: 2.25–3.14). For very preterm singleton birth, the median in the lowest SES group was 1.0 per 100 singleton live births across all countries (IQR: 0.87–1.12) compared to 0.6 per 100 live singleton births in the highest SES group (IQR: 0.59–0.66) (across all countries combined). Rates of moderate to late preterm singleton birth were again highest in the lowest SES group, with a median of 5.8 per 100 live singleton births across all countries (IQR: 5.27–6.40) compared to 4.4 per 100 live singleton births across all countries in the highest SES group (IQR: 4.13–4.65).

**TABLE 1:** Median, IQR and range for (1) rate of adverse outcome (for all births and by SES group), percentage excess adverse outcome associated with SES and concentration index across all countries and 2) the rate of adverse outcome for all births for countries with above or below median levels of concentration index.

		All countries						Countries with below median rate of adverse outcome	Countries with above median rate of adverse outcome
		Rate of adverse outcome				Percentage excess outcome	Concentration indices	Concentration indices	Concentration indices
Outcome	Measure	All births	Low SES	Mid SES	High SES				
Stillbirth per 1000 births	Median	3.42	4.88	3.32	2.65	21.3	−0.12	−0.12	−0.12
	Interquartile range	3.00 to 3.83	4.30 to 5.80	2.96 to 3.84	2.25 to 3.14	15.25 to 26.40	−0.15 to −0.10	−0.15 to −0.10	−0.15 to −0.10
	Range	2.50 to 4.37	3.36 to 8.62	2.13 to 5.70	1.47 to 3.87	−3.3 to 50.9	−0.28 to −0.01	−0.24 to −0.02	−0.28 to −0.01
Very preterm singleton birth per 100 live births	Median	0.78	0.96	0.80	0.64	17.9	−0.10	−0.08	−0.10
	Interquartile range	0.70 to 0.81	0.87 to 1.12	0.74 to 0.86	0.59 to 0.66	14.90 to 22.35	−0.13 to −0.07	−0.11 to −0.07	−0.13 to −0.08
	Range	0.58 to 0.94	0.74 to 1.36	0.57 to 1.24	0.43 to 0.78	6.7 to 27.0	−0.16 to −0.04	−0.14 to −0.06	−0.16 to −0.04
Moderate and late preterm singleton birth per 100 live births‐≥ 32 weeks	Median	4.97	5.76	4.99	4.39	10.1	−0.07	−0.04	−0.06
	Interquartile range	4.40 to 5.23	5.27 to 6.40	4.61 to 5.51	4.13 to 4.65	6.30 to 13.58	−0.07 to −0.04	−0.07 to −0.03	−0.08 to −0.05
	Range	3.59 to 7.65	4.41 to 9.81	3.65 to 8.32	3.20 to 7.25	3.9 to 18.4	−0.13 to −0.03	−0.08 to −0.03	−0.12 to −0.02

*Note:* The percentage excess outcome is based on the calculation of expected outcomes if the adverse outcome rate in the highest SES group is applied across all groups. It is expressed as the difference between the observed and expected outcomes as a percentage of total observed events. The concentration index measures the concentration of adverse outcomes in disadvantaged and advantaged groups. Values range from −1 (higher burden in lower SES groups) to +1 (higher burden).

### 3.3 Percentage of Excess Adverse Outcome Associated With SES

The percentage of excess stillbirth associated with SES ranged between 3% in Northern Ireland (a small increase) and 51% in Portugal (Table S2) with a median excess of 21% (IQR: 15.25–26.40) (Table 1). The median percentage excess of very preterm birth was 18% (IQR: 14.90–22.35) ranging from 7% in the Netherlands to 27% in Ireland, and for moderate and late preterm birth was 10% (IQR: 6.30–13.58) ranging between 4% in Portugal and 18% in Slovakia.

### 3.4 Concentration Indices

**FIGURE 3:**
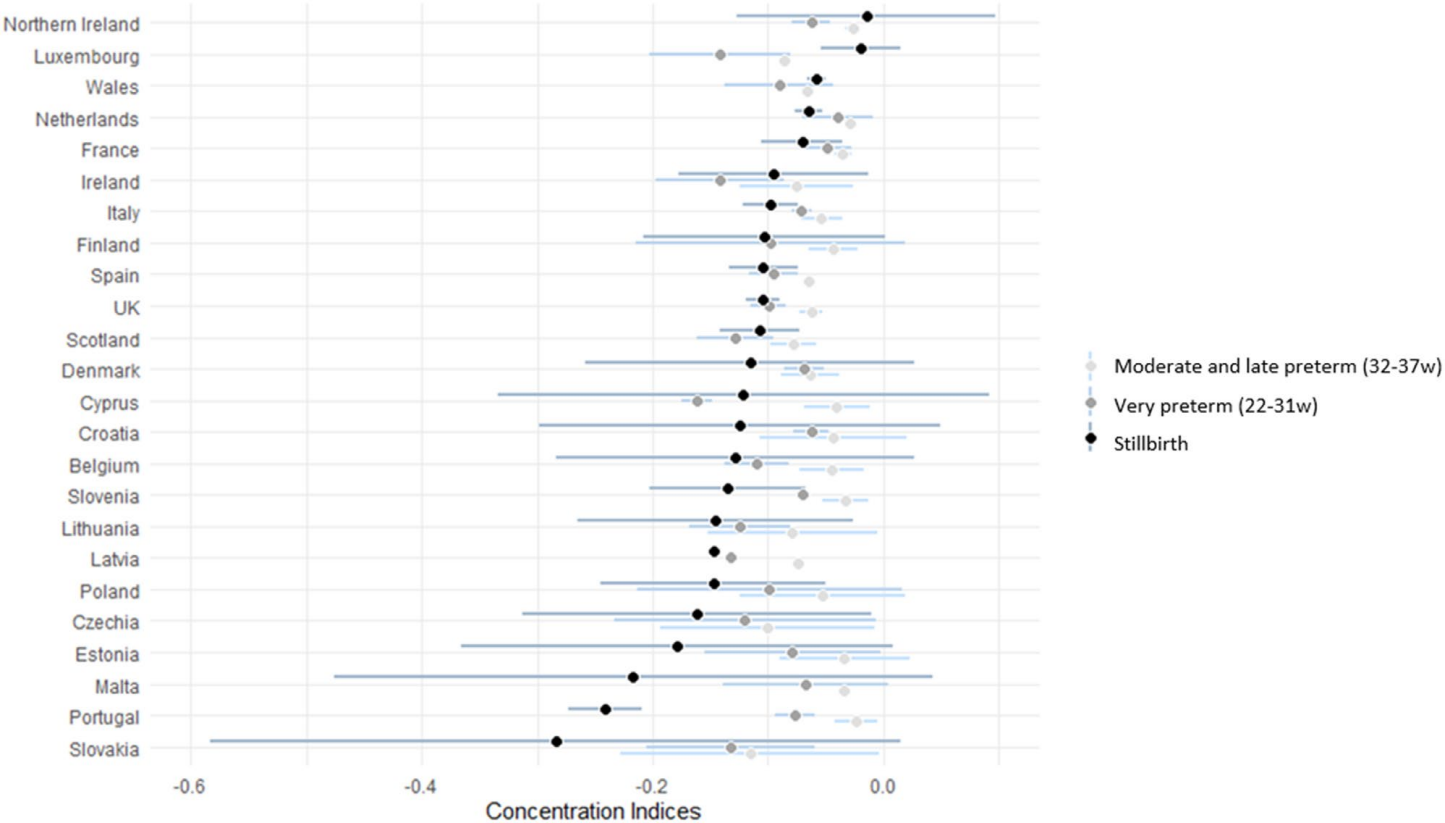
Concentration index of inequality by country for stillbirth rate per 1000 total births ≥ 24 weeks gestation, very preterm singleton birth rate per 100 singleton live births ≥ 22 weeks gestation and moderate and late preterm singleton birth rate per 100 singleton live births ≥ 32 weeks gestational age by country for period 2015–2019.

There was wide variation between countries in the CI for stillbirth, with a median of −0.12 (IQR: −0.15 to −0.10) and ranging between −0.01 and −0.28 (Table 1) (numbers nearer −1 reflecting greater inequality) with lowest inequality in Northern Ireland and highest in Slovakia to achieve equality (Data displayed graphically in Figure 3 and Cis by country detailed in table S3). For very preterm singleton birth, the median CI was −0.10 (IQR: −0.13 to 0.07) and ranged between −0.04 (Netherlands) and−0.16 (Cyprus). For moderate and late preterm singleton birth, the median CI was lowest at −0.07 (IQR: −0.07 to −0.04) and ranged between −0.03 (Portugal) and−0.13 (Slovakia). Figure 3 highlights how, generally, across most countries (16 out of 24) the CI showed higher levels of inequality for stillbirth compared to very preterm singleton birth and lowest levels of inequality for moderate and late preterm singleton birth.

### 3.5 Comparing Rates of Adverse Outcome With Concentration Indices

The distribution of the concentration index across countries with lower levels of adverse outcomes was very similar to those countries with higher rates of that outcome. The median CI (and IQR) for the 12 countries with below median levels of stillbirth was −0.12 (IQR: −0.15 to 0.10), the same as for those with higher levels of stillbirth (median−0.12, IQR: −0.15 to −0.10) (Table 1). For preterm birth patterns were similar (VPB: lower rate countries: median CI 0.08, IQR (−0.11 to −0.07); higher rate countries: median CI: −0.10, IQR (−0.13 to 0.08); MLPB: lower rate countries: median CI 0.04; (IQR: −0.07 to −0.03); higher rate countries: median CI −0.06 IQR (−0.08 to −0.05)). Associated Pearson correlation coefficients (PCC) supported a lack of association between the CI and rate for stillbirth (PCC = −0.019, *p* = 0.930), very preterm singleton birth (PCC = 0.063, *p* = 0.771) and moderate and late preterm singleton birth (PCC = 0.048, *p* = 0.825) (Figure S1).

## 4 Discussion

Our study findings reinforce the growing body of evidence indicating that socio‐economic inequalities play a significant role in shaping perinatal health outcomes. We used validated robust indicators of adverse perinatal outcomes and socio‐economic status across Europe to highlight widespread and varying socio‐economic inequalities for both stillbirth and preterm singleton birth across all participating countries, with higher levels of socio‐economic inequalities for more severe outcomes, very preterm birth and stillbirth compared with moderate and late preterm birth. We also found that similar levels of inequality were seen in countries with both higher and lower levels of adverse outcomes, suggesting the need to identify avoidable mortality to address inequalities within countries and improve maternal and newborn health.

## 5 Strengths and Limitations

The key strength of this study is the use of standardised routine population data with harmonised definitions to measure stillbirth and preterm birth rates among 17 million births over a 5‐year period from 24 countries. We mapped educational data to the ISCED‐97 classification to ensure consistent coding and discussed our findings with the countries involved for validation. By utilising the percentage excess adverse events and concentration indices, we were able to compare the inequalities in each country despite varying measures of SES across all 24 countries.

While our analysis provides an overall measure of existing inequalities in outcomes related to SES, due to the aggregated nature of the data received from the participating countries, we could not compare outcomes adjusted for individual characteristics such as mother's age [29, 30] or body mass index and smoking to identify those factors contributing to these gaps. This is an important area for further study which will be facilitated by Euro‐Peristat's common data model [16]. The level of missing data was higher for those babies with adverse perinatal outcomes, leading to underestimates of stillbirth and preterm singleton birth rates. We made an assumption that the probability of data being missing was unrelated to socio‐economic status. However, sensitivity analyses excluding those countries with high levels of missing data only suggested a mild change in the overall range of inequalities in health across included countries.

Sixteen countries were able to share perinatal outcomes by educational attainment. The SES distribution varied considerably between these countries, despite being based on the consistent ISCED‐97 classification [31]. This may reflect different educational attainment among the childbearing population in Europe, but despite our use of a standardised international classification, may also reflect the difficulty of creating homogenous groups that take into account different educational systems and, in particular, differential recognition of vocational versus academic qualifications across different routine data systems. By utilising additional measures of SES in addition to education, including occupation and area‐based deprivation, we were able to extend our study from 16 countries to include 24 out of the 31 countries in Euro‐Peristat. We recognise that aspects of SES measured varied across countries, particularly as education and occupation are measured at the individual level while the deprivation scores used were constructed for areas. These areas may not necessarily represent the individual‐level deprivation of residents living in the areas, especially in areas whose residents are diverse, including both more and less deprived individuals [32] and may underestimate inequality [33]. Research has also highlighted the importance of deprivation indices that are suitable for pregnant women when measuring inequalities in perinatal outcomes, as standard measures may underestimate this [34]. Despite these limitations, we identified substantial inequalities in all three adverse perinatal outcomes within all countries in this study. Further work exploring the inequalities observed using multiple measures available within individual countries that have both individual‐ and area‐level measures would progress understanding of the relationships between differing measures of SES and adverse outcomes, as education, area‐level deprivation and occupation may impact in different ways. For example, in some countries, women may have precarious jobs and poor material living conditions regardless of their educational level.

## 6 Interpretation

Our findings of socio‐economic inequalities in a range of perinatal health outcomes are in line with Patel et al. [35] work in low‐ and lower middle‐income countries. However, they found a similar SES gradient for stillbirth, perinatal and neonatal mortality, while we found that inequalities were greater for the more severe outcomes of stillbirth and very preterm singleton birth than moderate and late preterm birth. This may suggest that there are differences in Europe in the contribution of wider social determinants and health care to these outcomes [20, 36] and the role of social policies that diminish socio‐economically disadvantaged individuals' exposure to risk factors [11, 37].

The greater inequalities seen for stillbirth compared to preterm birth might suggest differences in mechanisms. Women of lower SES are reported as having a higher burden of stillbirth, but the mechanisms explaining this disparity remain unclear. In Sweden, the increased risks could not be explained by social differences in maternal age, height, body mass index, cigarette smoking or country of birth [38]. However, they found that term antepartum and intrapartum stillbirth were the deaths most associated with low social class, and these deaths may have been the most potentially preventable stillbirths. Countries such as Spain have shown these inequalities to be sustained, with a lack of improvement in stillbirth rates among women of low SES from 2007 – 2015 [39]. A recent systematic review concluded that the substantial socio‐economic inequalities in preterm birth are only partly explained by six groups of mediators [maternal smoking, maternal mental health, maternal physical health (including BMI), maternal lifestyle (including alcohol consumption), health care and working and environmental conditions], particularly maternal smoking in pregnancy. There is, however, a large residual direct effect of SES evident in most studies. In order to have a real impact on socio‐economic inequalities in perinatal health, policies need to identify gaps better.

We did not find any association between rates of adverse outcomes and levels of inequality in perinatal health across the 24 countries. This may suggest differences in national strategies to reduce poor perinatal outcomes or possibly how SES is measured. Strategies focused on the entire population may lower overall rates but can lead to widening socio‐economic inequalities, while national targeted interventions focusing on disadvantaged groups may reduce inequalities but have less impact on overall rates. The findings presented here can be used to identify potential countries with optimal interventions to improve both overall rates and inequalities.

## 7 Conclusion

Our study uses large‐scale national routine data to illustrate consistent associations between SES and adverse perinatal health outcomes across Europe. These associations were stronger for the more severe outcomes such as stillbirth, followed by very preterm birth, and highlight the impact of inequality across and within these countries. This work represents a key step on a pathway towards understanding contextual and policy factors associated with reduced inequalities to identify effective action to improve outcomes for all women and children, regardless of their socio‐economic status, and prevent avoidable mortality in future. This work documents the feasibility of routine monitoring of inequalities internationally, which is critical for evaluating strategies addressed at social determinants of health to reduce perinatal health disparities. It provides guidance for work to explore differences in measuring SES by educational level and by area‐level deprivation scores to improve comparability of between‐country variation in inequalities in health in the future. Such work needs to be combined with insights gained through talking to those with lived experience [40] to achieve significant steps towards health equity.

## Author Contributions

J.Z., L.S. and M.P. conceived and designed the study. M.P. compiled and prepared the study data; M.P. and L.S. conducted the analyses. J.Z. and M.P. had full access to all the data in the study. L.S., J.Z. and M.P. had final responsibility for the decision to submit the article for publication. All authors contributed to the interpretation of the results, participated in writing the manuscript, critically reviewed the manuscript and approved the final version for submission.

## Conflicts of Interest

The authors declare no conflicts of interest.

## Supporting information


Data S1.

## Data Availability

The data that support the findings of this study are available from the corresponding author upon reasonable request.
