# Supplementary material for: Socio‐Economic Inequalities in Stillbirth and Preterm Birth Rates Across Europe: A Population‐Based Study
Source: BJOG. 2025 Jul 10;132(13):2168–76. doi: 10.1111/1471-0528.18274 (PMC12592754; doi:10.1111/1471-0528.18274)
Supplement: Supplementary file 1 — Data S1. [file BJO-132-2168-s001.docx]

**Supplementary Box 1: Calculation of concentration indices**

The concentration index is calculated by ranking individuals from lowest to highest socioeconomic status and plotting the cumulative proportion of the population (e.g. births) against the cumulative proportion of the adverse outcomes (e.g. stillbirths). The concentration index measures twice the area between this curve and the line of equality. A concentration index of zero indicates equality, while negative values indicate stillbirths are disproportionately affecting lower SES groups.


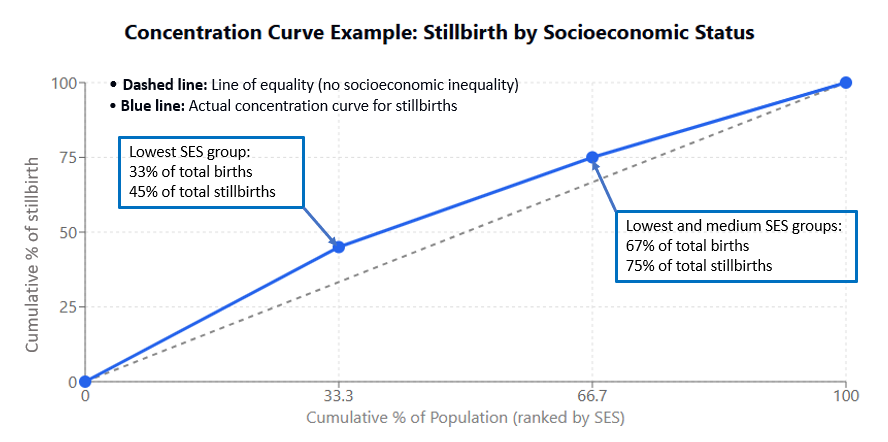


Concentration indices were calculated using the conindex command (1) in Stata [version 17], which implements the method described by Kakwani et al. (2) and calculates the CI using:

CI = (2/μ) × Cov(y, R)

Where:

- μ (mu) = mean of the outcome variable (stillbirth rate)
- y = outcome variable for each individual
- R = fractional rank of the SES variable (ranges from 0 to 1) calculated as the midpoint of the group (Here, lowest SES group has fractional rank 0.17)
- Cov = covariance

References

1. O'Donnell O, O'Neill S, Van Ourti T, Walsh B. conindex: Estimation of concentration indices. Stata J. 2016 1st Quarter;16(1):112-138. PMID: 27053927; PMCID: PMC4819995.
2. Kakwani N, Wagstaff A, van Doorslaer E. Socioeconomic inequalities in health: Measurement, computation, and statistical inference. Journal of Econometrics. 1997;77:87–103.

**Supplementary table S1: Number of births and percentage of missing socioeconomic status data for stillbirth ≥24 weeks gestation, very preterm singleton birth (22-31 weeks gestation) and moderate and late preterm singleton birth (32-36 weeks gestation) by country for period 2015-2019**

| Country | SES measure | Births (n) | | | | | % births missing SES group | | | | |
| --- | --- | --- | --- | --- | --- | --- | --- | --- | --- | --- | --- |
|  |  | Live birth | Stillbirth | Singleton live birth | | | Live birth | Stillbirth | Singleton live birth | | |
|  |  | ≥24^+0^ weeks | ≥24^+0^ weeks | 22^+0^-31^+6^ weeks | 32^+0^-36^+6^ weeks | All ≥22^+0^ weeks | ≥22^+0^ weeks | ≥24^+0^ weeks | 22^+0^-31^+6^ weeks | 32^+0^-36^+6^ weeks | All ≥22^+0^ weeks |
| Belgium | Education | 588762 | 2574 | 4470 | 32137 | 569449 | 12.1% | 62.7% | 17.9% | 13.2% | 12.1% |
| Croatia | Education | 180790 | 753 | 1192 | 7684 | 174988 | 28.4% | 31.9% | 41.3% | 29.6% | 28.2% |
| Cyprus | Education | 47238 | 155 | 359 | 3412 | 44964 | 0.7% | 0.6% | 0.6% | 0.6% | 0.7% |
| Czechia | Education | 551502 | 1734 | 4387 | 27039 | 535160 | 12.2% | 18.0% | 21.0% | 14.8% | 12.1% |
| Denmark | Education | 305064 | 767 | 2072 | 14431 | 295376 | 0.6% | 1.3% | 0.8% | 0.8% | 0.6% |
| Estonia | Education | 68120 | 187 | 455 | 2451 | 67034 | 0.0% | 0.0% | 0.0% | 0.0% | 0.0% |
| Finland | Education | 252996 | 633 | 1367 | 9457 | 235356 | 0.0% | 0.0% | 0.0% | 0.0% | 0.0% |
| France | Deprivation | 3653748 | 13841 | 27289 | 165820 | 3496348 | 2.7% | 4.3% | 4.0% | 3.2% | 2.6% |
| Ireland | Occupation | 310988 | 1192 | 1933 | 12118 | 299570 | 4.7% | 7.8% | 7.0% | 5.1% | 4.7% |
| Italy | Education | 2270455 | 6470 | 16036 | 110504 | 2194480 | 2.3% | 3.9% | 2.7% | 2.4% | 2.3% |
| Latvia | Education | 101783 | 447 | 797 | 3749 | 98986 | 0.0% | 0.0% | 0.0% | 0.0% | 0.0% |
| Lithuania | Education | 135666 | 524 | 865 | 4704 | 131848 | 0.0% | 0.0% | 0.0% | 0.0% | 0.0% |
| Luxembourg | Education | 34950 | 113 | 195 | 1689 | 33642 | 6.6% | 16.8% | 10.8% | 8.5% | 6.7% |
| Malta | Education | 22259 | 82 | 170 | 1027 | 21499 | 4.9% | 15.9% | 11.8% | 6.3% | 5.0% |
| Netherlands | Deprivation | 824308 | 2386 | 6681 | 35176 | 797553 | 0.6% | 0.7% | 1.0% | 0.7% | 0.6% |
| N. Ireland | Deprivation | 117160 | 442 | 859 | 5810 | 113642 | 0.5% | 0.2% | 0.9% | 0.4% | 0.5% |
| Poland* | Education | 761156 | 2489 | 14388 | 92686 | 1865682 | 1.4% | 2.9% | 1.1% | 0.7% | 0.6% |
| Portugal | Education | 433376 | 1298 | 3207 | 22461 | 419252 | 5.7% | 43.5% | 5.1% | 4.4% | 5.7% |
| Scotland | Deprivation | 258234 | 923 | 2210 | 14396 | 250223 | 0.1% | 0.7% | 0.5% | 0.2% | 0.1% |
| Slovakia | Education | 285852 | 1159 | 2301 | 14055 | 278779 | 13.1% | 15.0% | 15.3% | 13.8% | 13.1% |
| Slovenia | Education | 98841 | 260 | 725 | 4413 | 95294 | 3.0% | 7.3% | 4.4% | 3.1% | 3.0% |
| Spain | Education | 1720062 | 5167 | 12651 | 78830 | 1608925 | 8.0% | 16.0% | 9.8% | 8.1% | 7.0% |
| UK | Deprivation | 3637706 | 13900 | 32757 | 184631 | 3526426 | 0.4% | 0.3% | 0.3% | 0.3% | 0.4% |
| Wales | Deprivation | 143089 | 624 | 1105 | 6527 | 117667 | 2.0% | 1.4% | 3.1% | 2.4% | 1.5% |
| TOTAL | N/A | 16804105 | 58120 | 138471 | 855207 | 17272143 | 3.6% | 8.4% | 4.4% | 3.6% | 3.2% |

*Data only available for 2018-2019 for stillbirth in Poland, respective live births ≥24+0 weeks also provided for 2018-2019

** Terminations of pregnancy were excluded from stillbirth rates because of their different aetiology except for Belgium, Cyprus and the Netherlands where this information was not available

Supplementary table S2 Adverse outcome rates by SES group and percentage of excess adverse outcome associated with SES (95% CI) for stillbirth (per 1000 total births >=24 weeks), very preterm singleton birth (per 100 singleton live births >= 22 weeks gestation) and moderate and late preterm singleton birth (per 100 singleton live births ≥32 weeks gestation)

|  | Stillbirth | | | | Very preterm birth | | | | Moderate and late preterm birth | | | |
| --- | --- | --- | --- | --- | --- | --- | --- | --- | --- | --- | --- | --- |
|  | Low SES | Mid SES | High SES | **% excess assoc. with SES** | Low SES | Mid SES | High SES | **% excess assoc. with SES** | Low SES | Mid SES | High SES | **% excess assoc. with SES** |
| **Belgium** | 4.95  (4.53 to 5.42) | 5.7  (5.39 to 6.02) | 3.04  (2.84 to 3.25) | 30.1%  (24.4% to 35.3%) | 1.01  (0.94 to 1.07) | 0.9  (0.86 to 0.94) | 0.61  (0.58 to 0.64) | 21.9%  (17.3% to 25.9%) | 6.19  (6.03 to 6.36) | 6.14  (6.03 to 6.25) | 5.15  (5.06 to 5.24) | 9.5%  (7.6% to 11.1%) |
| **Croatia** | 8.62  (7.03 to 10.6) | 4.33  (3.95 to 4.75) | 3.14  (2.74 to 3.59) | 24.3%  (12.9% to 35.4%) | 0.8  (0.65 to 1) | 0.74  (0.69 to 0.8) | 0.57  (0.52 to 0.63) | 16.1%  (5.5% to 25.3%) | 6.13  (5.66 to 6.64) | 4.48  (4.35 to 4.62) | 4.05  (3.89 to 4.21) | 8.4%  (4.8% to 12.3%) |
| **Cyprus** | 7.41  (5.04 to 10.9) | 3.45  (2.43 to 4.91) | 2.81  (2.3 to 3.42) | 14.2%  (-9.6% to 34.2%) | 1.36  (1.02 to 1.82) | 1.24  (1.03 to 1.5) | 0.62  (0.54 to 0.72) | 21.7%  (6.7% to 33.9%) | 9.81  (8.76 to 11.0) | 8.32  (7.71 to 8.99) | 7.25  (6.96 to 7.56) | 5.2%  (0.5% to 9.6%) |
| **Czechia** | 4.81  (4.47 to 5.18) | 2.73  (2.5 to 2.97) | 2.33  (2.13 to 2.54) | 25.8%  (18.7% to 32.9%) | 1.15  (1.09 to 1.2) | 0.74  (0.7 to 0.78) | 0.66  (0.63 to 0.7) | 19.1%  (14% to 23.8%) | 6.79  (6.65 to 6.93) | 4.69  (4.59 to 4.79) | 4.27  (4.18 to 4.36) | 16.2%  (14.1% to 18.1%) |
| **Denmark** | 4.08  (3.51 to 4.75) | 2.5  (2.2 to 2.84) | 2.09  (1.88 to 2.32) | 16.2%  (4.6% to 26%) | 0.86  (0.79 to 0.93) | 0.75  (0.68 to 0.81) | 0.63  (0.59 to 0.67) | 10.8%  (4.6% to 16.9%) | 5.7  (5.51 to 5.9) | 5.55  (5.37 to 5.74) | 4.38  (4.28 to 4.48) | 11%  (8.6% to 13.4%) |
| **Estonia** | 5.44  (4.01 to 7.36) | 2.89  (2.33 to 3.6) | 1.95  (1.53 to 2.5) | 28.6%  (6.2% to 47.1%) | 0.74  (0.57 to 0.96) | 0.8  (0.7 to 0.91) | 0.56  (0.48 to 0.65) | 17.6%  (3.1% to 31%) | 4.53  (4.07 to 5.05) | 3.65  (3.43 to 3.89) | 3.51  (3.3 to 3.72) | 4.8%  (-2.1% to 11.3%) |
| **Finland** | 3.71  (3.15 to 4.38) | 2.49  (2.2 to 2.83) | 2.09  (1.84 to 2.37) | 16.3%  (4.5% to 28%) | 0.87  (0.78 to 0.97) | 0.57  (0.52 to 0.62) | 0.49  (0.45 to 0.54) | 14.9%  (6.2% to 23.5%) | 4.73  (4.5 to 4.97) | 4.15  (4.01 to 4.28) | 3.73  (3.61 to 3.85) | 7.8%  (4.4% to 11%) |
| **France** | 4.67  (4.51 to 4.82) | 3.81  (3.71 to 3.91) | 3.29  (3.2 to 3.38) | 12.8%  (10.1% to 15.5%) | 0.9  (0.88 to 0.93) | 0.79  (0.77 to 0.8) | 0.71  (0.7 to 0.72) | 9%  (6.9% to 11.1%) | 5.29  (5.23 to 5.34) | 4.86  (4.82 to 4.89) | 4.45  (4.42 to 4.49) | 6.9%  (6% to 7.7%) |
| **Ireland** | 4.98  (4.53 to 5.48) | 3.54  (3.2 to 3.91) | 3.22  (2.91 to 3.56) | 15.6%  (5.2% to 25.1%) | 0.9  (0.84 to 0.97) | 0.63  (0.58 to 0.68) | 0.47  (0.43 to 0.51) | 27%  (20.3% to 34.1%) | 5  (4.85 to 5.16) | 3.92  (3.8 to 4.04) | 3.53  (3.42 to 3.64) | 13.4%  (10% to 16.3%) |
| **Italy** | 3.6  (3.45 to 3.75) | 2.78  (2.68 to 2.89) | 2.22  (2.11 to 2.33) | 22%  (17.5% to 26.5%) | 0.85  (0.83 to 0.88) | 0.74  (0.72 to 0.76) | 0.6  (0.58 to 0.62) | 18.1%  (15.2% to 21.2%) | 5.83  (5.77 to 5.89) | 4.99  (4.95 to 5.04) | 4.48  (4.42 to 4.53) | 11.7%  (10.6% to 12.9%) |
| **Latvia** | 5.71  (5.08 to 6.42) | - | 3.14  (2.7 to 3.65) | 28.2%  (14.9% to 40%) | 1.03  (0.94 to 1.12) | - | 0.6  (0.54 to 0.67) | 25.3%  (15.5% to 35.5%) | 4.41  (4.22 to 4.61) | - | 3.28  (3.12 to 3.44) | 14.2%  (9.5% to 19.4%) |
| **Lithuania** | 7.41  (5.94 to 9.25) | 4.65  (4.01 to 5.4) | 3.07  (2.73 to 3.46) | 20.2%  (7.6% to 31.6%) | 1.06  (0.88 to 1.28) | 0.82  (0.73 to 0.92) | 0.54  (0.49 to 0.59) | 18.1%  (8.8% to 26.9%) | 5.48  (5.03 to 5.97) | 3.96  (3.76 to 4.17) | 3.2  (3.08 to 3.33) | 10.8%  (6.7% to 14.8%) |
| **Luxembourg** | 3.58  (2.42 to 5.31) | 3.16  (2.16 to 4.61) | 3.17  (2.47 to 4.07) | 2.4%  (-30.8% to 30.2%) | 0.78  (0.59 to 1.02) | 0.74  (0.58 to 0.95) | 0.43  (0.35 to 0.54) | 24.9%  (2.7% to 43.1%) | 6.36  (5.76 to 7.02) | 5.61  (5.11 to 6.16) | 4.34  (4.04 to 4.65) | 14.1%  (6.9% to 21%) |
| **Malta** | 6.06  (4.55 to 8.08) | 2.13  (1.1 to 4.09) | 2.51  (1.71 to 3.69) | 31.6%  (-9.3% to 58.5%) | 0.95  (0.75 to 1.2) | 0.71  (0.49 to 1.02) | 0.71  (0.57 to 0.9) | 10.2%  (-17.8% to 31.6%) | 5.21  (4.71 to 5.78) | 4.83  (4.19 to 5.57) | 4.51  (4.1 to 4.96) | 6.3%  (-4.6% to 15.7%) |
| **Netherlands** | 3.36  (3.09 to 3.65) | 3.06  (2.87 to 3.25) | 2.48  (2.32 to 2.66) | 14%  (6.3% to 20.5%) | 0.96  (0.91 to 1.01) | 0.83  (0.8 to 0.87) | 0.78  (0.75 to 0.81) | 6.7%  (2.8% to 10.9%) | 4.83  (4.72 to 4.95) | 4.52  (4.45 to 4.6) | 4.18  (4.11 to 4.26) | 6%  (4.2% to 7.8%) |
| **N. Ireland** | 4.37  (3.63 to 5.24) | 3.32  (2.85 to 3.87) | 3.87  (3.32 to 4.52) | -3.3%  (-22.4% to 15.1%) | 0.87  (0.76 to 0.99) | 0.79  (0.72 to 0.88) | 0.65  (0.57 to 0.73) | 14.9%  (2.6% to 25.2%) | 5.48  (5.19 to 5.78) | 5.25  (5.04 to 5.46) | 4.83  (4.61 to 5.05) | 6.3%  (1.7% to 10.6%) |
| **Poland*** | 6.11  (5.43 to 6.89) | 3.87  (3.66 to 4.09) | 2.45  (2.3 to 2.6) | 25%  (19.6% to 30.2%) | 1.34  (1.28 to 1.41) | 0.84  (0.82 to 0.86) | 0.65  (0.63 to 0.67) | 15.7%  (13.1% to 18.1%) | 7.17  (7.01 to 7.34) | 5.19  (5.14 to 5.25) | 4.6  (4.55 to 4.64) | 8.2%  (7.3% to 9.2%) |
| **Portugal** | 4.69  (4.31 to 5.09) | 3.43  (3.13 to 3.74) | 1.47  (1.3 to 1.66) | 50.9%  (43.8% to 57.4%) | 0.92  (0.87 to 0.98) | 0.77  (0.73 to 0.82) | 0.65  (0.61 to 0.69) | 14.9%  (9.2% to 20.7%) | 5.8  (5.66 to 5.95) | 5.32  (5.19 to 5.45) | 5.19  (5.08 to 5.3) | 3.9%  (1.5% to 6.3%) |
| **Scotland** | 4.67  (4.17 to 5.22) | 3.56  (3.21 to 3.95) | 2.79  (2.47 to 3.16) | 21.6%  (10.2% to 32.2%) | 1.2  (1.12 to 1.29) | 0.89  (0.83 to 0.95) | 0.65  (0.6 to 0.71) | 26.3%  (19.4% to 32.9%) | 7.07  (6.86 to 7.29) | 5.83  (5.68 to 5.99) | 4.88  (4.74 to 5.03) | 15.9%  (13.3% to 18.5%) |
| **Slovakia** | 8.22  (7.57 to 8.93) | 3.15  (2.83 to 3.52) | 2.26  (2 to 2.55) | 44%  (36.5% to 51.5%) | 1.17  (1.09 to 1.26) | 0.81  (0.76 to 0.87) | 0.63  (0.59 to 0.68) | 23.7%  (17.1% to 29.7%) | 7.19  (6.99 to 7.41) | 4.7  (4.56 to 4.84) | 4.15  (4.03 to 4.27) | 18.4%  (15.7% to 21%) |
| **Slovenia** | 4.41  (3.1 to 6.28) | 3.06  (2.57 to 3.65) | 2  (1.65 to 2.43) | 23.4%  (4.1% to 39.6%) | 0.95  (0.74 to 1.21) | 0.85  (0.76 to 0.95) | 0.66  (0.6 to 0.74) | 12.9%  (0.3% to 23.7%) | 5.5  (4.95 to 6.1) | 4.85  (4.63 to 5.08) | 4.4  (4.22 to 4.6) | 5.6%  (0.6% to 10.5%) |
| **Spain** | 3.82  (3.65 to 3.98) | 3.02  (2.83 to 3.21) | 2.46  (2.36 to 2.57) | 17.9%  (13.7% to 22%) | 0.96  (0.93 to 0.99) | 0.87  (0.83 to 0.9) | 0.65  (0.63 to 0.66) | 17.8%  (14.9% to 20.4%) | 5.73  (5.66 to 5.79) | 5.14  (5.06 to 5.23) | 4.36  (4.31 to 4.41) | 11.7%  (10.6% to 12.8%) |
| **UK** | 4.98  (4.82 to 5.15) | 4.02  (3.92 to 4.13) | 3.01  (2.92 to 3.1) | 21%  (18.2% to 23.6%) | 1.2  (1.18 to 1.23) | 0.98  (0.96 to 0.99) | 0.74  (0.73 to 0.76) | 20%  (18.3% to 21.7%) | 6.27  (6.21 to 6.33) | 5.46  (5.42 to 5.5) | 4.62  (4.59 to 4.66) | 12.5%  (11.8% to 13.3%) |
| **Wales** | 5.02  (4.36 to 5.8) | 4.37  (3.87 to 4.93) | 3.8  (3.27 to 4.4) | 12.9%  (-2.4% to 27.2%) | 1.11  (1 to 1.24) | 1.01  (0.92 to 1.1) | 0.71  (0.63 to 0.8) | 24.1%  (14.8% to 33.5%) | 6.53  (6.24 to 6.83) | 5.7  (5.49 to 5.93) | 4.73  (4.51 to 4.96) | 15.5%  (10.9% to 19.7%) |

**Supplementary table S3: Rate, concentration index (CI) for stillbirth per 1000 total births ≥24 weeks gestation, very preterm singleton birth per 100 singleton live births ≥22 weeks gestation and moderate and late preterm singleton birth per 100 singleton live births ≥32 weeks gestational age by country for period 2015-2019**

|  | **Stillbirth** | | **Very preterm birth** | | **Moderate and late preterm birth** | |
| --- | --- | --- | --- | --- | --- | --- |
|  | **Rate** | **Concentration Index** | **Rate** | **Concentration Index** | **Rate** | **Concentration Index** |
| Belgium | 4.4 | -0.13 (0.027,-0.284) | 0.78 | -0.11 (-0.081,-0.137) | 5.7 | -0.05 (-0.016,-0.073) |
| Croatia | 4.2 | -0.13 (0.049,-0.298) | 0.68 | -0.06 (-0.048,-0.077) | 4.4 | -0.04 (0.020,-0.107) |
| Cyprus | 3.3 | -0.12 (0.092,-0.334) | 0.8 | -0.16 (-0.148,-0.175) | 7.6 | -0.04 (-0.012,-0.069) |
| Czechia | 3.1 | -0.16 (-0.010,-0.313) | 0.82 | -0.12 (-0.007,-0.233) | 5.1 | -0.10 (-0.008,-0.194) |
| Denmark | 2.5 | -0.12 (0.027,-0.259) | 0.70 | -0.07 (-0.052,-0.086) | 4.9 | -0.06 (-0.038,-0.089) |
| Estonia | 2.7 | -0.18 (0.008,-0.366) | 0.68 | -0.08 (-0.002,-0.156) | 3.7 | -0.03 (0.022,-0.090) |
| Finland | 2.5 | -0.10 (0.002,-0.209) | 0.58 | -0.10 (0.019,-0.215) | 4.0 | -0.04 (-0.023,-0.065) |
| France | 3.8 | -0.07 (-0.035,-0.106) | 0.78 | -0.05 (-0.027,-0.069) | 4.8 | -0.04 (-0.028,-0.043) |
| Ireland | 3.8 | -0.10 (-0.013,-0.177) | 0.65 | -0.14 (-0.086,-0.197) | 4.1 | -0.08 (-0.027,-0.125) |
| Italy | 2.8 | -0.10 (-0.074,-0.122) | 0.73 | -0.07 (-0.062,-0.079) | 5.1 | -0.05 (-0.036,-0.071) |
| Latvia | 4.4 | -0.15 (-0.147,-0.147) | 0.81 | -0.13 (-0.132,-0.132) | 3.8 | -0.07 (-0.074,-0.074) |
| Lithuania | 3.9 | -0.15 (-0.026,-0.265) | 0.66 | -0.13 (-0.081,-0.168) | 3.6 | -0.08 (-0.006,-0.152) |
| Luxembourg | 3.2 | -0.02 (0.015,-0.054) | 0.58 | -0.14 (-0.080,-0.203) | 5.0 | -0.09 (-0.084,-0.088) |
| Malta | 3.7 | -0.22 (0.042,-0.476) | 0.79 | -0.07 (0.004,-0.139) | 4.8 | -0.03 (-0.029,-0.040) |
| Netherlands | 2.9 | -0.06 (-0.052,-0.077) | 0.84 | -0.04 (-0.009,-0.069) | 4.4 | -0.030 (-0.026,-0.033) |
| N Ireland | 3.8 | -0.15 (0.098,-0.127) | 0.76 | -0.06 (-0.046,-0.079) | 5.2 | -0.03 (-0.020,-0.033) |
| Poland | 3.3 | -0.15 (-0.050,-0.245) | 0.77 | -0.10 (0.016,-0.213) | 5.0 | -0.05 (0.018,-0.124) |
| Portugal | 3.0 | -0.24 (-0.209,-0.273) | 0.76 | -0.08 (-0.059,-0.094) | 5.4 | -0.02 (-0.005,-0.042) |
| Scotland | 3.6 | -0.11 (-0.073,-0.142) | 0.88 | -0.13 (-0.095,-0.162) | 5.8 | -0.08 (-0.058,-0.098) |
| Slovakia | 4.1 | -0.28 (0.014,-0.583) | 0.83 | -0.13 (-0.059,-0.205) | 5.1 | -0.12 (-0.004,-0.228) |
| Slovenia | 2.6 | -0.14 (-0.067,-0.203) | 0.76 | -0.07 (-0.070,-0.070) | 4.7 | -0.03 (-0.013,-0.053) |
| Spain | 3.0 | -0.10 (-0.074,-0.134) | 0.79 | -0.10 (-0.075,-0.116) | 4.9 | -0.06 (-0.065,-0.065) |
| UK | 3.8 | -0.10 (-0.090,-0.119) | 0.93 | -0.10 (-0.085,-0.115) | 5.3 | -0.06 (-0.052,-0.072) |
| Wales | 4.4 | -0.06 (-0.050,-0.066) | 0.94 | -0.09 (-0.043,-0.138) | 5.6 | -0.07 (-0.063,-0.069) |

**Supplementary Figure S1: Rate of adverse outcome versus concentration index by country with linear trend and Pearson correlation coefficient for stillbirth rate per 1000 total births ≥24 weeks gestation, very preterm singleton birth rate per 100 singleton live births ≥22 weeks gestation and moderate and late preterm singleton birth rate per 100 singleton live births ≥32 weeks gestational age by country for period 2015-2019**

| Stillbirth per 1000 births >=24 weeks gestation: Pearson correlation coefficient=-0.019, p=0.930 | Very preterm singleton birth per 100 singleton births >= 22 weeks gestation: Pearson correlation coefficient=0.063, p=0.771 | Moderate and late preterm singleton birth per 100 singleton births >=32 weeks gestation: Pearson correlation coefficient=0.048, p=0.825 |
| --- | --- | --- |
| 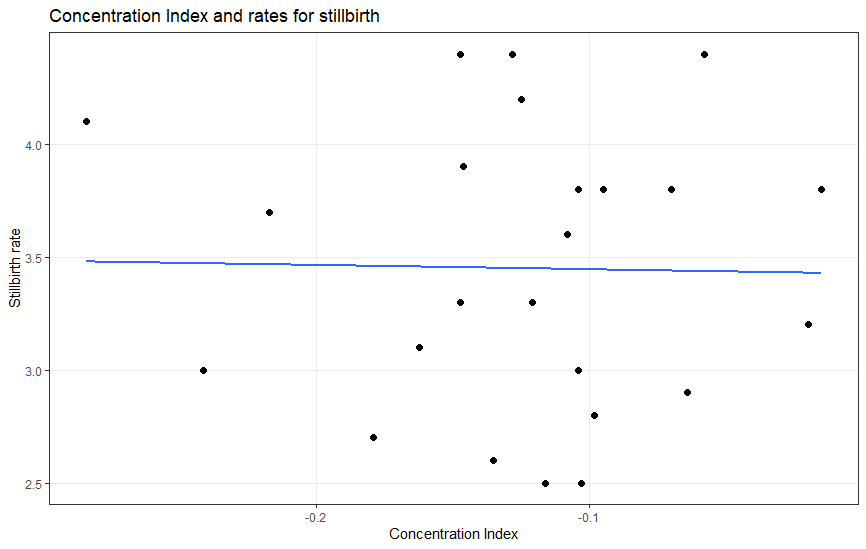 | 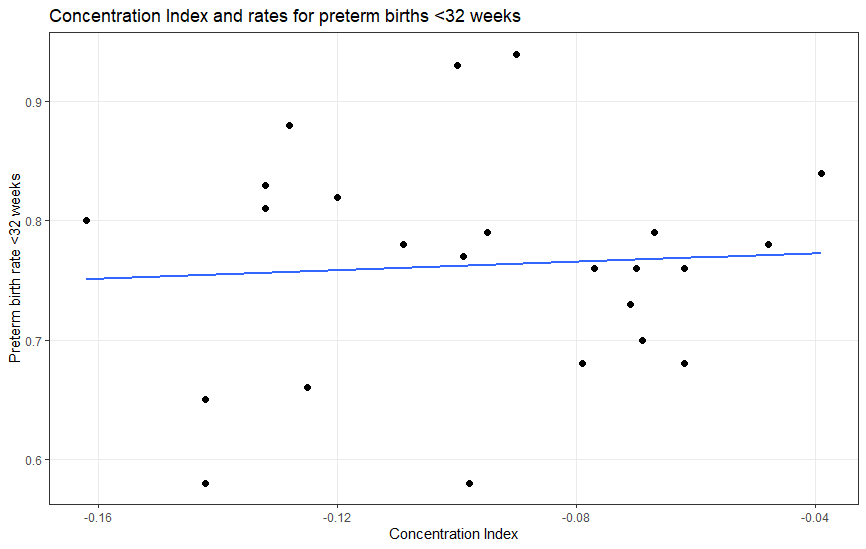 | 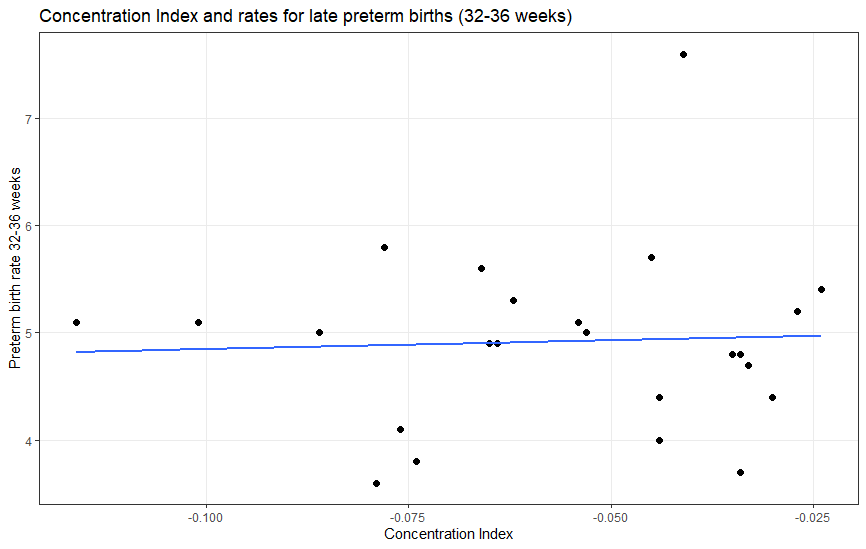 |
